# The diagnostic challenge of non-traumatic bladder rupture: a pictorial essay

**DOI:** 10.1007/s11604-023-01395-1

**Published:** 2023-02-02

**Authors:** Hideki Kunichika, Junko Takahama, Hidehiko Taguchi, Masayo Haga, Emiko Shimoda, Masayoshi Inoue, Kengo Morimoto, Nagaaki Marugami, Toshihiro Tanaka

**Affiliations:** 1 Department of Diagnostic and Interventional Radiology, Nara Medical University, 840, Shijo-cho, Kashihara, , Nara 634-8522 Japan; 2 Department of Radiology, Higashiosaka City Medical Center, 3-4-5 Nishiiwata, Higashiosaka, Osaka 578-8588 Japan

**Keywords:** Bladder rupture, Non-traumatic, Computed tomography, Pseudo-renal failure

## Abstract

Non-traumatic bladder rupture (NTBR) is relative rare pathology including spontaneous rupture and iatrogenic injury. As increasing the medical intervention for the pelvic malignancy or elderly population, NTBR will be encountered more frequently. There are few previous studies summarizing the imaging features of NTBR. We reviewed imaging characteristics of 18 previous cases of NTBR experienced. In addition, 3 presentative cases that can be a pitfall to differentiate from NTBR. The aim of this article is to clarify the key CT findings of NTBR and its pitfalls.

## Introduction

There are two types of bladder rupture (BR): traumatic and non-traumatic. The former is more common associated with abdominopelvic traumatic injuries, and 1.6% of these injuries represent BR [1, 2]. The non-traumatic type is rare including spontaneous rupture and iatrogenic injury. Spontaneous rupture is caused by vulnerability and hyperextension of the bladder wall such as subsequent radiotherapy, binge alcohol drinking and neurogenic bladder [3]. Iatrogenic injury can be associated with gynecological and colorectal surgery and urologic procedures (transurethral resection of the bladder tumor (TURBT) and Foley catheter placement). BR is a rare event, with reported incidences of only 0.002% in the emergency department setting [4] and 2.0% following radiotherapy for cervical cancer [5]. BR as complications of TURBT occurs with an incidence of 0.9–5% [6]. As increasing the medical intervention for the pelvic malignancy or elderly population, non-traumatic bladder rupture (NTBR) will be encountered more frequently. Computed tomography (CT) findings of NTBR are often non-specific and there are few previous studies summarizing the imaging features. We aim to provide the CT findings of NTBR through this pictorial essay.

## Clinical characteristics

We identified 18 NTBR proved clinically in affiliated 2 institutions from December 2005 to February 2022. The patients’ characteristics are summarized in Table 1. Typically, BR represents lower abdominal pain or various symptoms including hematuria, dysuria and anuria [7]. In our case series, the patients were often complained of non-specific symptoms; abdominal pain (83%), following fever (37.5 °C or higher), abdominal distension and urinary retention. It is clinically important to differentiate unexpected NTBR from other acute abdomen by CT findings [8].

**Table 1:** Patient characteristics and clinical symptoms (*n* = 18)

Characteristics	
Age; median (range)	69 (40–88) years old
Sex: male/female	7/11
Clinical symptoms	*n* (%)
Abdominal pain	15 (83%)
Fever	3 (17%)
Abdominal distension	3 (17%)
Urinary retention	2 (11%)
Septic shock	1 (6%)
None	1 (6%)

The causes of BR are shown in Table 2. Post-TURBT for bladder cancer and post-irradiation for gynecological malignancy were the most frequent. Tanaka et al. reported in the study of 97 patients that the common cause of spontaneous BR was after radiotherapy (58%), followed by neurogenic bladder (17%) [9].

**Table 2:** The estimated causes of bladder rupture (*n* = 18)

The estimated causes	*n*
Post TURBT for superficial bladder cancer	5
Post irradiation for vaginal cancer or uterine cervical cancer	5
Urinary retention associated with diabetes mellitus	2
Post-operative laparoscopy for appendicitis	1
Bladder tamponade with renal hemorrhage	1
Bladder hemangioma with Klippel–Trenaunay–Weber syndrome	1
Unknown	3

### CT findings

We classified the CT findings of NTBR into two categories: direct and indirect findings. Direct ones are discontinuity of bladder wall and leakage of contrast medium from the bladder. Indirect ones are extra-luminal gas in peritoneal space, ascites, retroperitoneal fluid collection or gas, and hydronephrosis. CT images were evaluated with thin slice [1 mm] axial images and multiple plane reconstruction (MPR). Contrast-enhanced CT (CECT) was performed in 6 of 18 cases. A dose of 80–100 mL of nonionic iodinated contrast medium (300–370 mgI/mL) was administered intravenously at 1–3 mL/seconds with an automated injector system followed by acquisition of images in 2–3 phases. Delayed phase imaging was obtained in all CECT patients at 150–180 s after injection of contrast medium. In the three of 6 CECT cases, additional super delayed phases (2, 6, 15 h later) were performed. The collected data have been summarized in Table 3.

**Table 3:** Direct and indirect CT findings in patients with non-traumatic bladder rupture (*n* = 18)

Findings	*n*
Direct findings	
Discontinuity of bladder wall	7/18 (39%)
Leakage of contrast medium from the bladder	3/6 (50%) *
Initial routine study (delayed phase (150–180 s later))	0/6 (0%)
Additional study (super delayed phase (2,6,15 h later))	3/3 (100%) *
Indirect findings	
Ascites	14/18 (78%)
Reached to the surface of the liver as large ascites	11/14 (79%)
Extra-luminal gas in peritoneal space	6/18 (33%)
Retroperitoneal fluid collection / gas	11/18 (61%)
Perivesical and/or prevesical space	11/18 (61%)
Above the perivesical space	2/11 (18%)
Hydronephrosis	7/18 (39%)
Clinical finding	
Pseudo-renal failure	8 / 14 (57%) **

*CECT was performed in 6 of 18 cases. Additional super delayed phase was scanned in 3 of 6 cases

**We excluded 1 patient on dialysis and 3 patients with incomplete blood test records

### Direct findings

#### Discontinuity of bladder wall (Figs. 1, 2, 3)

**Fig. 1:**
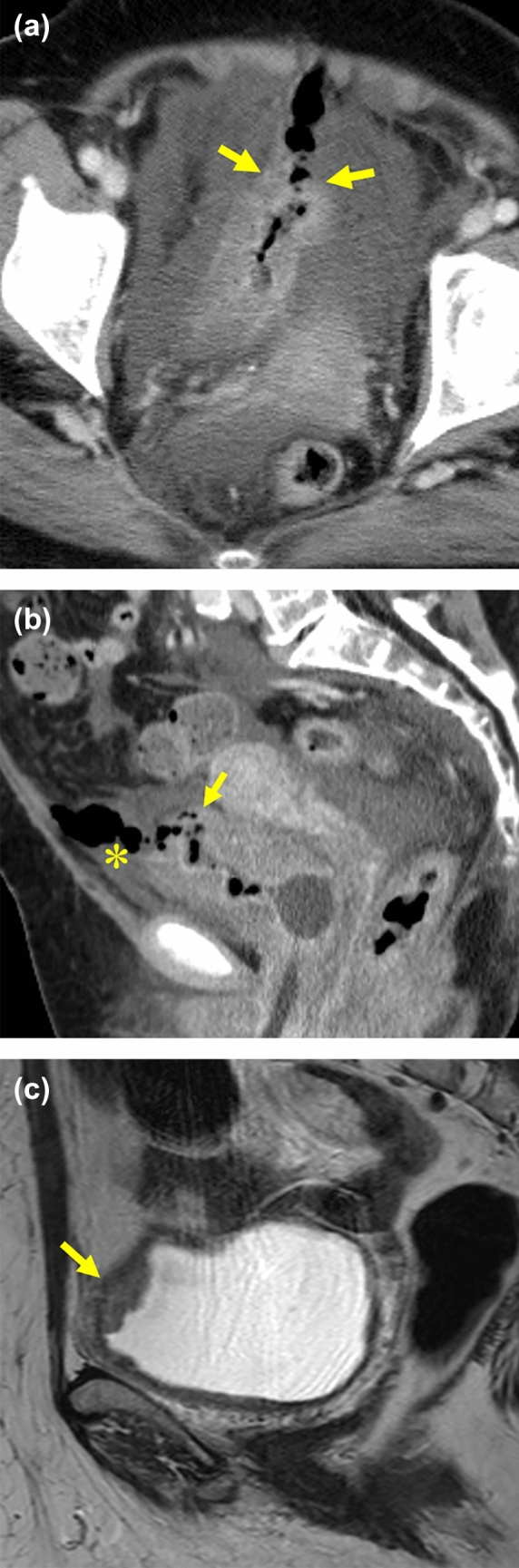
60-Year-old woman with bladder rupture complained abdominal pain the day after TURBT.** a**,** b** The axial a and sagittal b images of CECT show the tract-like gas at the bladder dome (arrows), retroperitoneal fluid collection with gas (in the paravesical space and Retzius cavity) (*) and moderate amount of ascites.** c** T2-weighted MR image before TURBT shows bladder tumor matching with the ruptured point (arrow)

**Fig. 2:**
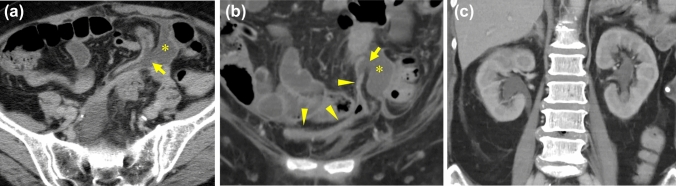
60-Year-old woman with bladder rupture who also had bacteremia, lung abscess, and epidural abscess of the lumbar spine. The cause of this rupture was unknown.** a b** The axial a and MPR.** b** CECT images demonstrate bladder wall (arrowheads) and its discontinuity (arrows). Small amount of uremic ascites (*) leaks through the ruptured site.** c**. Bilateral hydronephrosis is also observed as the indirect finding

**Fig. 3:**
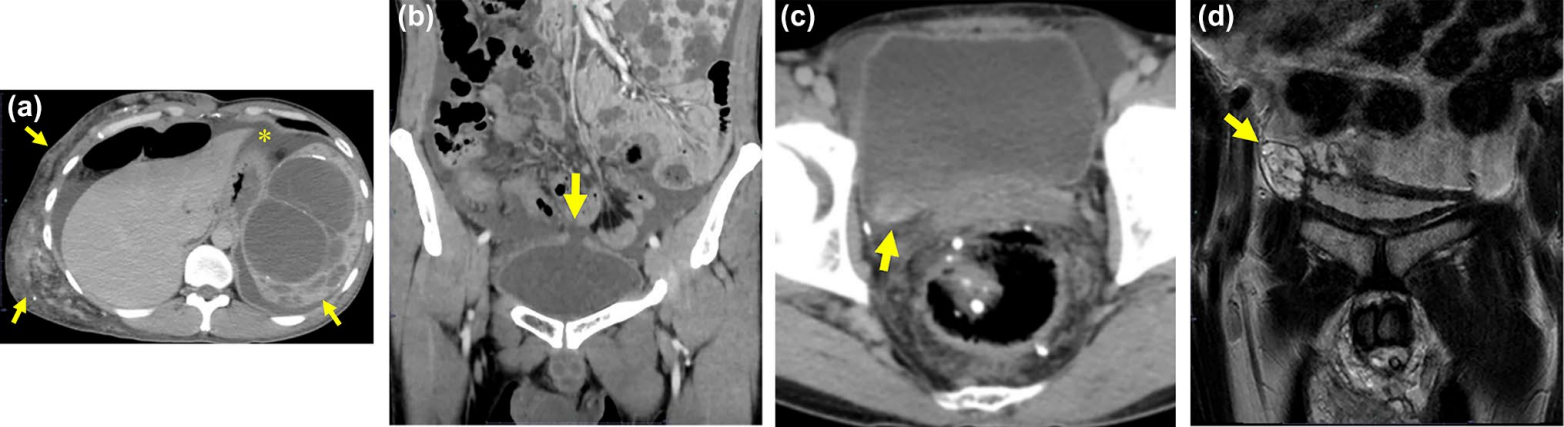
40-Year-old male with bladder rupture due to bladder hemangioma associated with Klippel-Trenaunay-Weber syndrome. He presented with lower abdominal pain and pseudo-renal failure.** a** CECT shows massive ascites (*) and lymphangioma of subcutaneous and spleen (arrows) associated with Klippel–Trenaunay–Weber syndrome.** b** The coronal image shows the discontinuity of bladder wall (arrow) and low dense uremic ascites.** c** Small amount of contrast medium has reached to the bladder (arrow) in delayed phase (180 s after administration of contrast medium) but the uremic ascites showed still low density (*). It is difficult to identify the leakage of contrast medium.** d** T2-weighted MR image shows bladder hemangioma as the cause of bladder hemorrhagic tamponade in the past (arrow). The fragility of the bladder wall may be caused by the history of tamponade and several placements of catheter

To observe the spherical three-dimensional structure of the bladder requires thin slices with multiple directions. Though we reviewed the CT images with thin slices from multiple directions, the discontinuity of bladder wall was recognized only in 39%. We present three cases showed the discontinuity of bladder wall. This finding is obviously specific but also low sensitivity to diagnose BR because the ruptured point is obscure in the collapsed bladder.

#### Leakage of contrast medium from the bladder (Figs. 4, 5)

**Fig. 4:**
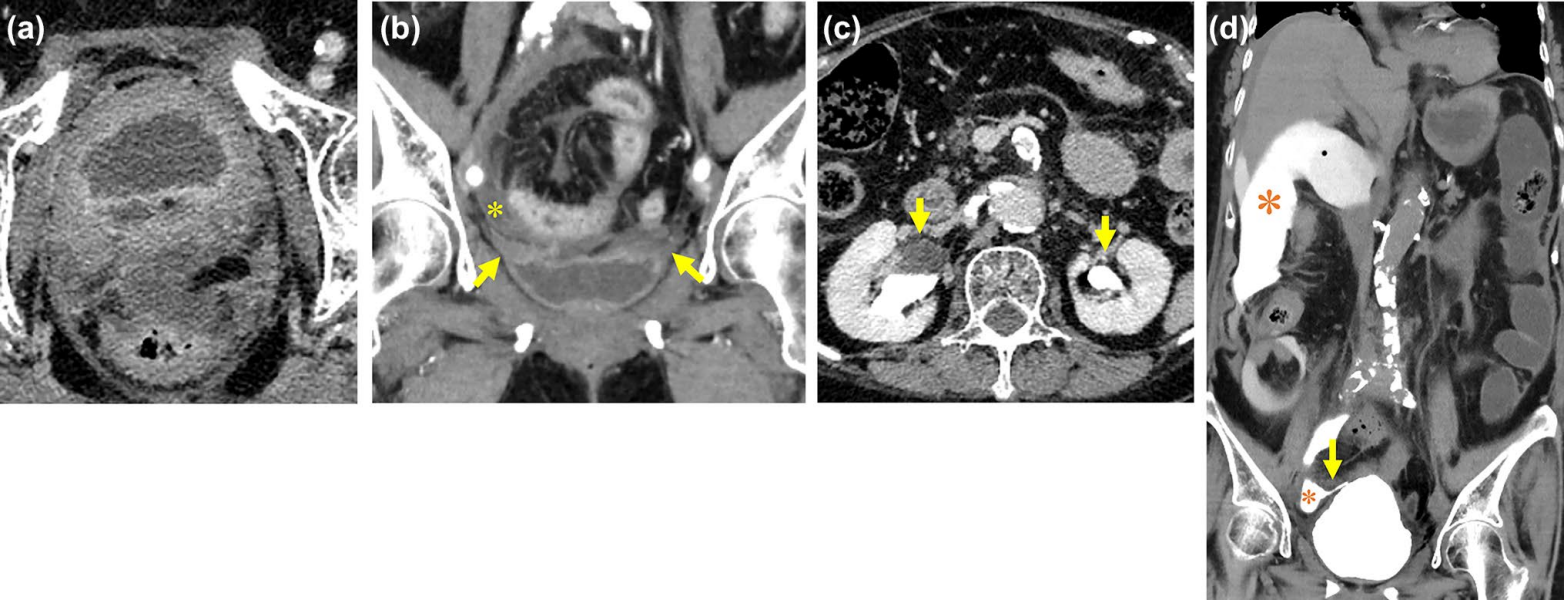
80-Year-old woman with bladder rupture, who presented lower abdominal pain and abdominal distension. She had the history of radiation therapy for cervical cancer 27 years ago.** a b** CECT in axial a and coronal** b** images shows thickened bladder wall without any discontinuity. Retroperitoneal fluid (arrow) and a small amount of ascites (*) are observed but no leakage of contrast medium.** c** Bilateral hydronephrosis is also represented.** d** The additional super delayed phase obtained the next day shows leakage of contrast medium through the pin-hale of the bladder (arrow) predominantly on the right side of the abdominal cavity (arrowhead)

**Fig. 5:**
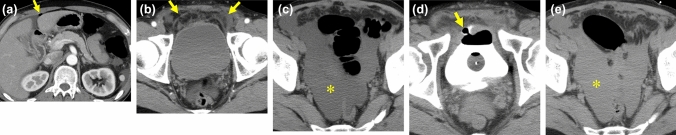
50-Year-old man with iatrogenic bladder rupture (post-operative laparoscopy for appendicitis), who presented with abdominal pain and abdominal distension three days after laparoscopic appendicectomy.** a** CECT shows small amount of extra-luminal gas suitable for postoperative state.** b** The bladder wall keeps continuity though prevesical fluid collection is observed (arrows).** c** A large amount of ascites (*) represents low attenuation in delayed phase.** d e** The additional super delayed phase (2 h later) shows clearly the perforation point (arrow) and increased value of ascites (approximately 10 HU to 50 HU) (*), which reflects leakage of contrast medium

CECT was performed in 33.3% (6/18), and no leakage of contrast medium was observed (0/6) in delayed phase obtained 150–180 s after injection of contrast medium. Though Deck et al. reported that the sensitivity and the specificity for diagnosing intraperitoneal BR using CT cystography were 78 and 99%, respectively [10], no leakage could be detected in routine CECT series. In the three of 6 cases, additional super delayed phase taken several hours later revealed the leakage of contrast medium into the abdominal cavity (Fig. 4, 5). The routine CECT delayed scan was too early to observe leakage of contrast medium. If NTBR is suspicious from the clinical information or routine CECT, the additional CT scan after few hours later as the super-delayed phase can be the key feature.

### Indirect findings

#### Ascites (Fig. 5)

A large amount of uremic ascites was reported as specific in traumatic BR [11]. In our NTBR cases, ascites was seen in 78% (14/18), and had reached to the liver surface in 79% (11/14). Though the uremic ascites was also reported as relative low density (< 10 HU) [11], only 9 of 18 cases (50%) showed low dense ascites (< 10HU) in our cases. A large amount of ascites reached to the liver surface is relative specific findings at NTBR, but the density of ascites can vary.

#### Extra-luminal gas in peritoneal space (Figs. 5, 6)

**Fig. 6:**
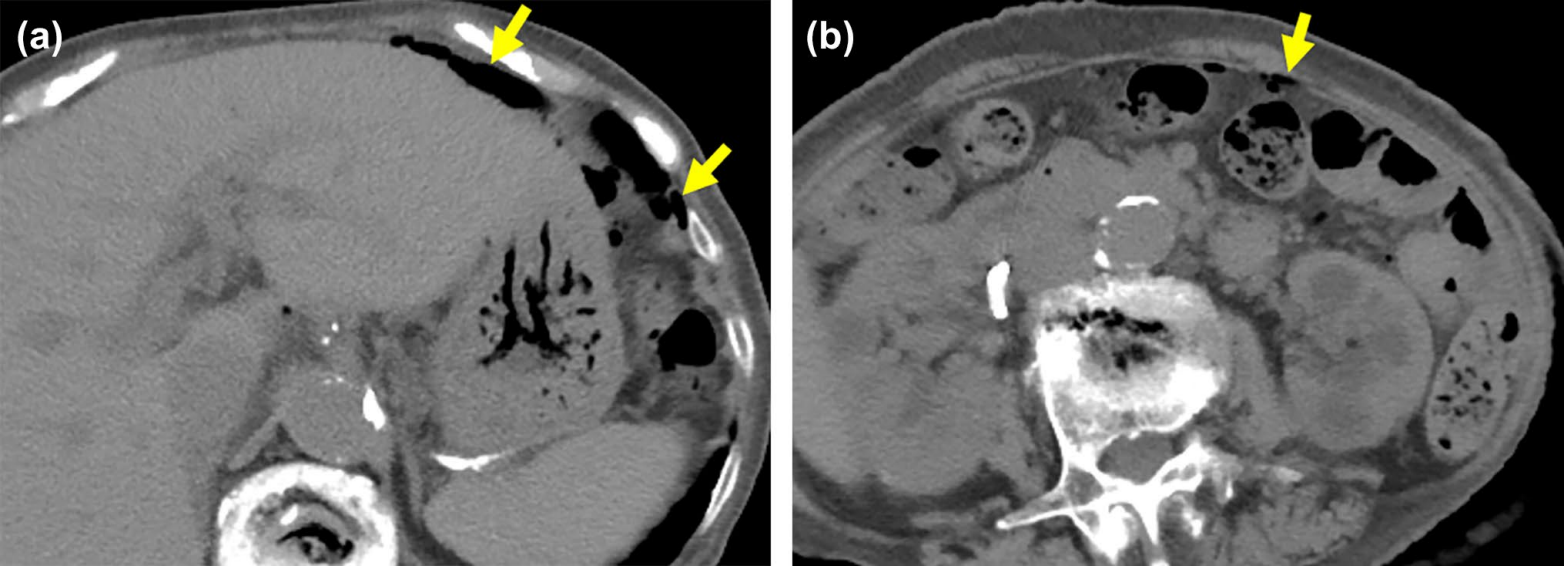
80-Year-old woman complaining acute abdomen for bladder rupture with a long-term indwelling bladder catheter and a right ureteral stent for chronic kidney disease.** a b** NECT shows only extra-luminal gas (arrows) and no other direct or indirect findings of bladder rupture. An exploratory laparotomy was performed for suspected small bowel perforation, and it revealed bladder rupture due to catheter injury and a small amount of infected ascites in the Douglas pouch. Peritonitis caused by infected urinary ascites is difficult to distinguish from small bowel perforation clinically and radiologically

Extra-luminal gas in peritoneal space was seen in 33.3%, which is spreading from the dissected bladder wall suggests us of the possibility of BR. In the case of BR in Fig. 6, only extra-luminal gas was observed as the sign of BR. This may be due to the backflow from the Foley catheter tip directly and drainage of uterine from the bladder, respectively. The differential diagnosis of extra-luminal gas is perforation of the gastrointestinal tract that is much more common than BR.

#### Retroperitoneal fluid collection or gas (Figs. 1, 4)

Retroperitoneal fluid collection or gas in perivesical and/or prevesical space was observed in 61% (11/18), and had reached above the perivesical space in 2/11 (18%). The fluid collection predominantly in the pelvic retroperitoneum was not specific but can be the key finding for NTBR.

#### Hydronephrosis (Figs. 2, 4)

Hydronephrosis was seen in 39%. Of 2 patients with BR had history of radiotherapy for cervical cancer. Miyauchi et al. reported that hydronephrosis as a late effect was observed in 1.3% of cervical cancer patients treated with CCRT or RT after surgery [12]. Ureteral reflux and insufficient compliance of urinary bladder wall are important factor for NTBR associated with hydronephrosis, especially in the radiation cystitis.

### Clinical analysis—pseudo-renal failure caused by reverse autoperitoneal dialysis

**Fig. 7:**
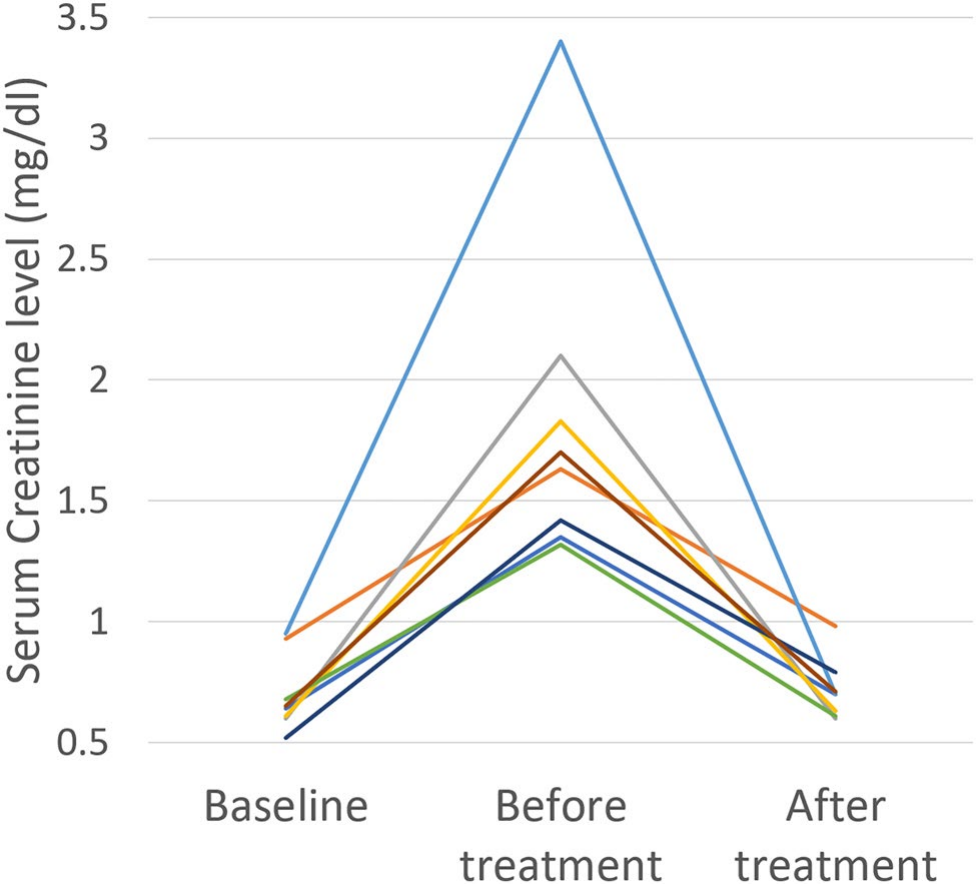
Transient increase of serum creatinine levels (> 0.5 mg/dL) was detected in 8/14 patients (57%), excluding 1 patient on dialysis and 3 patients with incomplete blood test records

The transient increase of the serum creatinine levels caused by uroperitoneum refers to “Pseudo-renal failure”. This increase is explained by “reverse autoperitoneal dialysis [13]. This phenomenon is characterized small molecules including creatinine in uroperitoneum transferred across the peritoneal membrane into the blood stream. Transient increase of serum creatinine levels (> 0.5 mg/dL) was detected in 8/14 patients (57%), excluding 1 patient on dialysis and 3 patients with incomplete blood test records (Fig. 7). The creatinine levels were decreased immediately after therapy for NTBR in all 8 cases. Although there may have been some other effects, such as dehydration, we consider it is likely that the phenomenon of reverse autoperitoneal dialysis was occurring.

### Pitfalls

#### Perforation of the small intestine (*Fig. **8*)

**Fig. 8:**
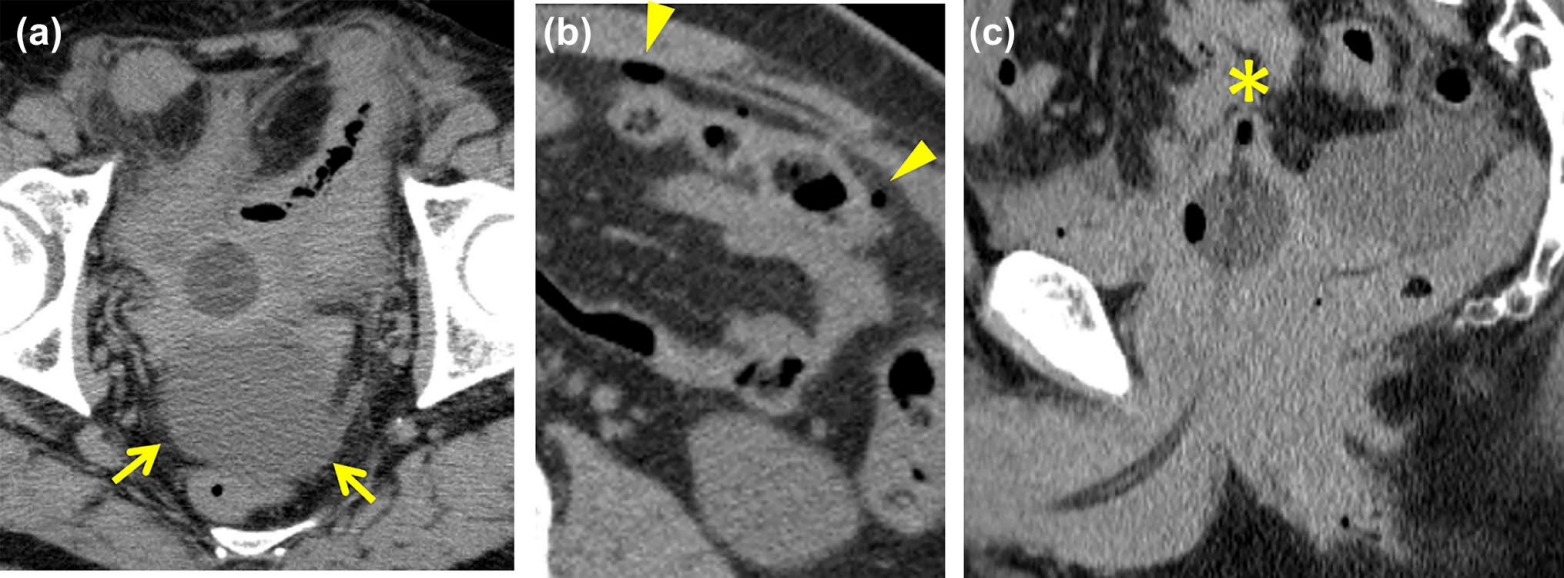
Pitfalls. 70-Year-old man with perforation of the small intestine who presented with urinary retention and abdominal pain.** a b** Axial NECT images obtained after Foley catheter placement demonstrate ascites (arrows) and extra-luminal gas (arrowheads). The CT value of ascites is relatively high (25-30HU).** c** A sagittal image reveals thinning of the bladder wall around the catheter tip (*), which led to a misdiagnosis of bladder rupture. Subsequent cystography was obtained and ruled out bladder rupture. Exploratory laparotomy revealed perforation of the small intestine.

Perforation of the small intestine is more common and represents similar CT findings. Mizumura et al. reported that BR should be considered when the ascites showed less than 10 HU [8]. CT values of ascites were not specific, but a massive low dense ascites reached to the liver surface can be the key to differentiate BR from small bowel perforation.

#### Emphysematous cystitis (*Fig. **9*)

**Fig. 9:**
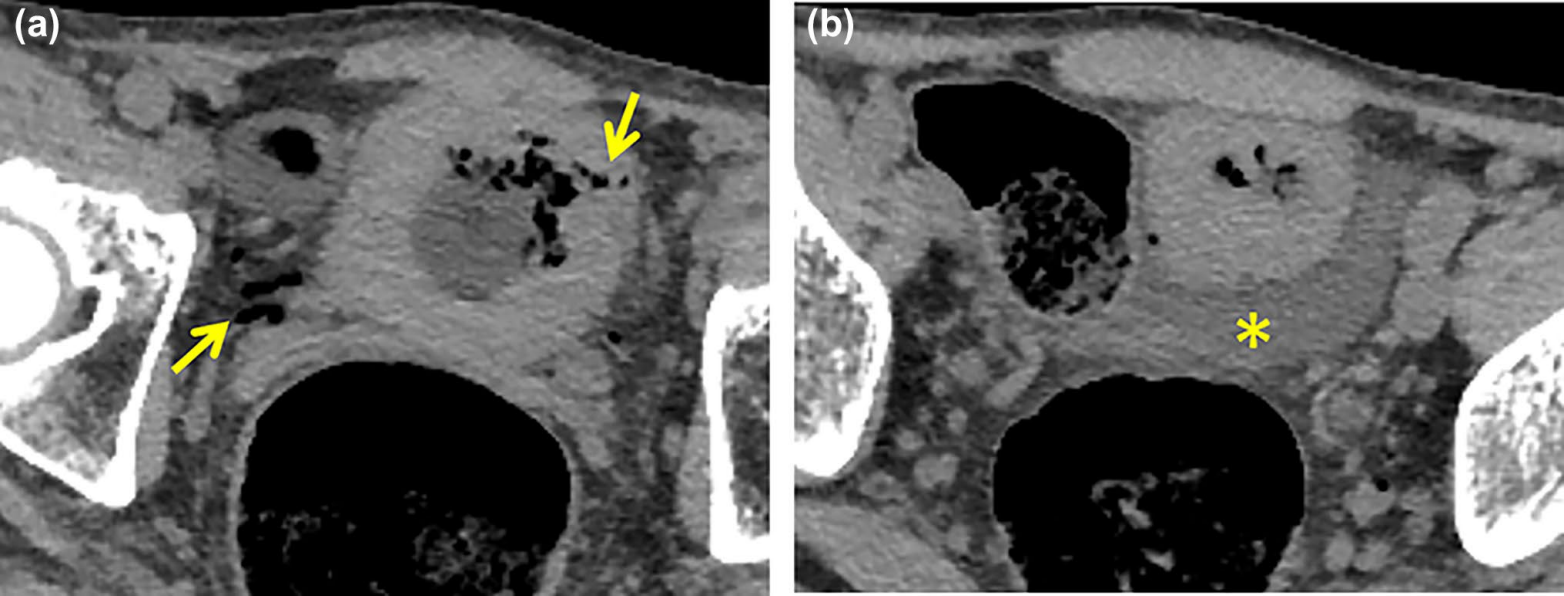
Pitfalls. 70-Year-old man with emphysematous cystitis who presented with fever and decreased appetite. He had a medical history of diabetes mellitus. **a b** NECT images show diffuse bladder wall thickening and linear intramural/retroperitoneal gas (arrows) and ascites (*). This patient was treated conservatively with insertion of bladder catheter. The penetrating gas of emphysematous cystitis can resemble that of a bladder wall fistula

Emphysematous cystitis is a potentially life-threatening bacterial infectious disease [14]. The intramural gas of bladder wall sometimes shows linear and penetrating through to the perivesical space. This appearance may be misdiagnosed as bladder wall fistula. However, unlike BR, emphysematous cystitis show only gas around the bladder without fluid retention.

#### Spontaneous uterine perforation with pyometra (*Fig. **10*)

**Fig. 10:**
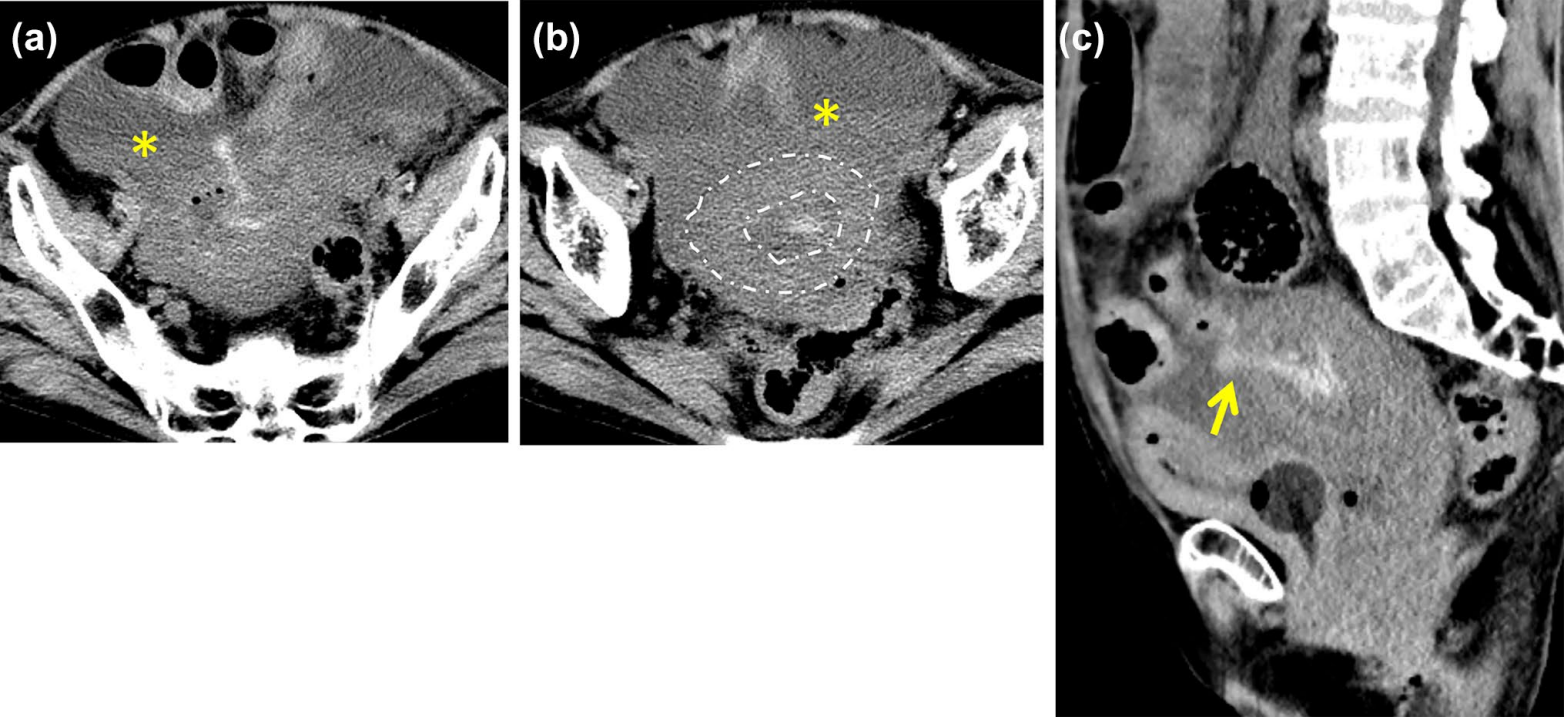
Pitfalls. 60-Year-old woman with acute peritonitis associated with perforation of pyometra uteri who presented with abdominal pain and septic shock.** a b** NECT image shows a large amount of relatively high intense ascites with an irregular hyperintensity in the pelvis (*). b Slightly high dense structure surrounding the heterogeneous hyperintensity appears to be the uterine myometrium (dot line) containing hemorrhage.** c** A sagittal reconstructed image shows the perforated point at the uterine fundus as an irregularly shaped high-density area (arrow). No extra-luminal gas can be identified in this case

Uterine perforation with pyometra is rare but increasing cause of acute abdomen in elderly bedridden women [15]. CT shows small amount of extraluminal gas and ascites and resembles BR or intestinal perforation. For the ascites from pyometra contains viscous pus, it tends to show higher density than uremic ascites. Enlarged uterine cavity may be also observed.

## Summary

The CT findings of NTBR are often non-specific in clinical situations and its differential diagnosis is challenging. The discontinuity of bladder wall is decisive direct findings but low sensitivity and the routine contrast-enhanced delayed scan was too early to detect the leakage of contrast medium. To diagnose NTBR, massive low intensity ascites, intraperitoneal extra-luminal gas, retroperitoneal fluid collection or gas and hydronephrosis can be the additional key indirect findings. When NTBR is suspected from these indirect findings or clinically, additional CT study as super delayed phase or direct cystography should be recommended. The pseudo-renal failure was also characteristics clinical feature and may often observed.

## Abbreviations

NTBR: Non-traumatic bladder rupture
BR: Bladder rupture
TURBT: Transurethral resection of the bladder tumor
MPR: Multi-planar reconstruction
CT: Computed tomography
CECT: Contrast-enhanced computed tomography
NECT: Non-enhanced computed tomography
HU: Hounsfield units

## Declarations

### Inform concern

The authors declare that they have no grants.
